# Examining Single Session Peer-Teaching Instructional Approaches on Pre-Service Physical Education Teachers’ Throwing Techniques

**DOI:** 10.1177/00315125231214126

**Published:** 2023-11-24

**Authors:** Bradley Beseler, Mandy S. Plumb, Michael Spittle, Nicola F. Johnson, Jack T. Harvey, Christopher Mesagno

**Affiliations:** 1 Institute of Education, Arts and Community, 1458 Federation University Australia, Ballarat, VIC, Australia; 2 School of Health, Medical and Applied Sciences, 527828 Central Queensland University, Cairns, QLD, Australia; 3 Institute for Health and Sport, 5399 Victoria University, Melbourne, VIC, Australia; 4 School of Education, 2498 Edith Cowan University, Joondalup, WA, Australia

**Keywords:** throwing, teaching style, reciprocal teaching, video analysis, peer teaching

## Abstract

An important role of a Physical Education (PE) teacher is to assist students to develop the fundamental motor skills (FMS) that will allow them to participate in physical activities with competence and confidence. Thus, PE teachers require the knowledge and skills to carry out this crucial task. In the crowded curricula of Physical Education Teacher Education (PETE) programs, there are limited opportunities for pre-service PE teachers to learn how to analyze and perform a large list of motor skills. Our purposes in this study were to determine whether a single session peer-teaching intervention could improve pre-service PE teachers’ short-term non-dominant hand overarm throwing performances and to examine these students’ perceptions of the interventions. We allocated 47 pre-service PE teaching students (24 males; 23 females) to one of three experimental groups: a Video Analysis Group (VAG; *n* = 17), a Verbal Group (VG; *n* = 19), and a Control Group (CG; *n* = 11), based on the class in which they were enrolled. VAG and VG participants worked with a partner of their choice in reciprocal peer-teaching to improve each other’s non-dominant hand throwing technique. VAG and VG interventions were identical except that VAG participants accessed video analysis technology. CG participants completed unrelated course work that involved no overarm throwing activities. A single 20-minute session of peer teaching with video analysis feedback during practice led to rapid enhancements in non-dominant hand overarm throwing skills. While all three groups improved their performance by retention testing, participants in the VAG group improved most quickly. Participants in both the VAG and VG groups reported that their respective interventions improved their throwing and Qualitative Movement Diagnosis (QMD) skills. Based on these results, we suggest that PETE programs integrate peer-teaching and video analysis sessions into fundamental movement courses to accelerate students’ motor skill acquisitions.

## Introduction

One of the most important roles of a Physical Education (PE) teacher is to assist students to develop the fundamental motor skills (FMS) that will allow them to participate in physical activities with competence and confidence throughout their lives. It is challenging for PE teachers to provide the quantity and quality of feedback needed for learners’ skill acquisition in the limited time allotted within crowded curricula. However, researchers have shown that feedback can be enhanced through peer teaching (Hamlin, 2005) and, potentially further, by providing learners access to video analysis technology (Casey & Jones, 2011). For PE teachers to capably implement these approaches they must have developed necessary skills within Physical Education Teacher Education (PETE) programs.

### Peer Teaching

A common form of peer teaching is Mosston and Ashworth’s (2002) Reciprocal Teaching (RT) in which learners generally work in pairs (dyads) to improve each other’s skills. In RT, one student acts as the learner (or tutee) and performs the task while another acts as the observer (or tutor) and provides immediate and ongoing feedback, guided by a teacher-designed criteria sheet (or checklist). When instructed, the students swap roles and repeat the process. Peer teaching engages the learner and encourages higher-order thinking (Harris, 2009), enhancing the observer’s cognitive processing, motivation and attention directed to the task (Ensergueix & Lafont, 2010). Working in pairs to analyze each other’s techniques empowers students to take control of their own learning (Hamlin, 2005). Additionally, the structure of reciprocal peer teaching allows it to be combined efficiently with video analysis. Using video analysis in combination with peer teaching allows the learner to witness themselves performing the skill, while they simultaneously hear the observer describe the performance, dramatically increasing the quantity and quality of feedback to help them better understand their performance kinaesthetically (Hamlin, 2005).

The use of video replay to provide feedback is widespread in coaching and PE instruction, but limited research has detailed the effectiveness of video feedback as a means of accelerating skill acquisition (Potdevin et al., 2018; Spittle, 2021; Weir & Connor, 2009). Video analysis researchers (Beseler & Plumb, 2019; Darden, 1999; Darden & Shimon, 2000; Menickelli et al., 2000; Roberts & Brown, 2008) have shown that learners must be familiar with seeing themselves on screen and should practice and analyze video replays for multiple sessions to achieve significant performance improvement. While Rothstein (1980) stated that a minimum of five sessions over a semester is required for significant improvement, this amount is impractical for each FMS PE teachers must acquire over a semester. Cognisant of the time pressures PE teachers face, we examined the effect of single session peer-teaching instruction with (and without) added video analysis technology on improving pre-service PE teachers’ overarm non-dominant throwing performance.

Motivation and confidence are important contributing factors to motor learning and performance and video analysis may have a positive impact on these learning variables (e.g., Darden, 1999; Ferracioli et al., 2013; Koh & Khairuddin, 2004; Kretschmann, 2017; O’Loughlin et al., 2013; Weir & Connor, 2009). Wulf and Lewthwaite’s (2016) OPTIMAL (Optimizing Performance Through Intrinsic Motivation and Attention for Learning) theory of motor learning suggested that confidence is a predictor of performance and self-efficacy. According to OPTIMAL, attentional and motivation factors contribute to performance and learning by strengthening the link between movement goals and actions. While Wulf and Lewthwaite (2016) identified research showing that video feedback enhanced the learning of swimming strokes and trampoline skills, limited further research has focused on how video analysis in single session learning might affect learners’ confidence and performance.

To investigate the effectiveness of two different single session peer-teaching instructional approaches to accelerate skill acquisition, we examined the fundamental motor skill of throwing with the non-dominant arm. Having a better understanding of the efficacy of these approaches may help pre-service PE teachers develop personal instructional skills to build FMS proficiency in their students more quickly. Previous research has identified significant gender differences in throwing performance, with males (relative to females) throwing more accurately, with higher velocity, and with a more developmentally advanced technique (e.g., Beseler et al., 2021; Johnson et al., 2020; Lorson et al., 2013). Considering the importance of comprehending the reasons behind the performance gap between males and females in this skill, we also sought to investigate the potential impact of gender on throwing performance.

In this study, we had five objectives: (a) to compare the effects on improving short-term overarm throwing performance of two different single session peer-teaching instructional approaches with a control group that experienced no peer-teaching or video analysis practice; (b) to ascertain whether peer-teaching with video analysis (versus without video analysis) better affected participants’ self-perceived abilities to perform and analyze the overarm throw in a single session; (c) to examine participants’ perceptions about the importance of video analysis in the Qualitative Movement Diagnosis (QMD) process; (d) to determine the impact of video analysis on participants’ enjoyment; and (e) to examine whether gender influenced throwing performance.

We hypothesised that, after the respective interventions (a) the Video Analysis Group (VAG) would throw with more advanced technique in post and retention testing, than the Verbal Group (VG), who would throw with more advanced technique than the Control Group (CG); (b) the VAG would have higher self-perceived ability to perform and analyze the overarm throw than the VG; (c) the VAG would report higher importance of the video analysis technology to the QMD process compared to the VG; and (d) the VAG would report their intervention to be more enjoyable than the VG; and (e) males would throw with a more advanced technique than females.

## Method

### Research Design

This study was conducted in an ecologically valid learning setting in a commonly available basketball court gymnasium (Ensergueix & Lafont, 2010; Miller-Cotto & Auxter, 2021). The activities conducted were representative of what students would normally do as part of their fundamental movement course. The activities, which could be easily implemented into PETE program, utilized a quasi-experimental between-subjects design with a sequence of pre-testing, intervention, post-testing, and retention testing. The pre-testing, intervention, and post-testing sessions were conducted during one of the participants’ scheduled fundamental movement classes. The retention testing occurred three weeks later in the same scheduled class.

### Participants

Participants were 47 university pre-service PE teaching students (*M*_*age*_ = 20.57; *SD* = 3.40 years) enrolled at the one campus in the same Bachelor of Health and Physical Education program (see Results section for power analysis). There were 24 males (*M*_*age*_ = 20.96; *SD* = 4.18 years) and 23 females (*M*_*age*_ = 20.09; *SD* = 2.33 years), who were naïve to the purposes of the study.

We conducted a power analysis to estimate a required sample size based on an examination of treatment-time interactions (i.e., differences between the changes over time in the three treatment groups) in a Repeated Measures Analysis of Covariance (RMANCOVA) using the throwing score, while covarying for gender and age. Based on data collected within previous throwing studies and fundamental movement class observations, we anticipated an initial value of 45 points for the adjusted mean of the throwing score at pre-test, and targets of six, three, and one point in the mean throwing score increase from pre- to post-test for the VAG, VG, and CG respectively, with mean reductions of one point in each group at retention. Based on over two decades of fundamental movement class observations and the data collected from previous throwing studies, we assumed a ‘within-groups’ standard deviation of four points, corresponding to a range of approximately 16 points due to individual differences between participants after accounting for gender and treatment differences. This resulted in a “medium” effect size (Cohen, 2013) of .24. Under the assumptions of constant correlation over time (sphericity), with a conservatively estimated magnitude of *r* = .7 (Mosher & Schutz, 1983), with a statistical significance level of 5% and 80% power, the required participant sample size calculated using GPower software (Faul et al., 2007) was 24 (i.e., three groups of 8). Focusing solely on the two intervention groups, the mean changes over time postulated for VAG and VG corresponded to a “small” to “medium” (Cohen, 2013) effect size of .17. For 80% power to detect a mean difference of this magnitude in a post hoc pairwise comparison, the required sample size increased to 36 (i.e., three groups of 12), for which our sample size of 47 was considered sufficient.

### Preliminary Ethical Procedures

All activities of the study were explained to participants, and all participants provided their informed consent prior to any engagement in study activities. The study was approved by the University Human Research Ethics Committee.

### Study Procedures

Prior to pre-testing, the three classes in which participants were enrolled each became one of the experimental groups: (a) VG (*n* = 19), (b) VAG (*n* = 17), and (c) CG (*n* = 11). All sessions were completed during the participants’ normally scheduled classes, decreasing the likelihood of feedback crossover (Jennings et al., 2013). A narrated video explaining the experimental procedures was shown to all groups prior to the pre-testing. To ensure that participants understood the overarm throw, we showed participants a throwing technique video, including footage of elite and proficient throwers accompanied by verbal narration and visual cueing outlining the six critical components of the throw on a 102 cm TV. These videos were chosen because Janelle et al., (2003) found that video modelling with verbal and visual cueing led to better technique acquisition and retention when learning a novel skill, and this method ensured that all participants received standardized instructions (Talpey et al., 2016).

The system chosen to measure throwing technique was a modified version of the developmental levels with over 40 years of research and cross-sectional and longitudinal validation (Beseler et al., 2021; Roberton & Halverson, 1984; Williams et al., 1998. (A copy of the developmental levels can be obtained from the corresponding author upon request). This component approach evaluated each body component on an ordinal developmental scale (e.g., 1–6), with higher levels representing more developmentally advanced movement patterns (Logan et al., 2017). Haywood et al. (2012) explained that the wide use of these levels has had robust developmental validity. This component approach provided a more precise description of the movement changes than other qualitative assessment systems (Roberton & Konczac, 2001).

### Pre-Testing

In pre-testing, all participant groups completed three non-dominant hand overarm throws because the task novelty would help control for throwing experience (Southard, 2006). Participants threw a tennis ball with maximum force, but they were advised that accuracy and velocity of the throws would not be measured.

We selected throwing technique as the primary dependent variable since it is a common, practical form of assessment often applied by PE teachers in class settings. In addition, throwing technique, is a process measure that is more accurate than product or outcome measures like speed and distance thrown that are influenced by noise variance in participant body size and strength (Haywood & Getchell, 2014).

Testing took place on a single court gymnasium at the first author’s university. Two Sony Cybershot DSC-RX100 Mark I cameras (Sony Corporation) with a shutter speed of 16.67 ms were placed on tripods at a height of 1.3 m to film individual throws at a rate of 50 frames/s. The camera on the open side (throwing arm side) was set up perpendicular to ball release and direction of the throw. The other camera was set up directly behind the line of the participant’s ball release (see Beseler et al., 2021). Both cameras were 5 m from thrower.

### Experimental Groups

VAG and VG participants worked in reciprocal pairs (Mosston & Ashworth, 2002) and the VAG had access to video analysis technology while the VG did not. The VAG and VG interventions involved one member of the pair throwing (thrower) for the first 5 min, whilst their partner (observer) provided feedback; roles were then reversed for the next 5 min. This process was repeated twice so that each participant threw for 10 min. Reciprocal observers (Ernst & Byral, 1998; Kretschmann, 2017) used the throwing component checklist (available from the corresponding author upon request) and QMD skills learned in earlier practical and theoretical classes to develop the thrower’s performance.

The checklist included text, photographs, and visual annotations. It helped observers analyze their partners’ throwing and focused observers’ attention on one component at a time (Koh & Khairuddin, 2004), guiding them as to which weaknesses to remedy first, consistent with the principle that PE teachers should provide remediation to help learners improve throwing (Knudson, 2013). Thus, each component on the checklist included a remedy that identified how common errors could be rectified. The remedies chosen for each component were based on corrections identified in the literature (e.g., Beseler & Plumb, 2019; Roberton & Halverson, 1984; Southard, 2006) and discovered by the first author’s teaching and coaching experience. During the VAG intervention, the VAG had access to the Hudl Technique video analysis smart phone app (Version 6.1.3.2; available from https://apps.apple.com/au/app/hudl-technique/id470428362). This app allowed participants to video record and analyze throwing performances. Participants used the Hudl Technique analysis app on several occasions prior to the intervention, so that they would not be distracted by the novelty of seeing themselves on screen for the first time (Beseler & Plumb, 2019; Darden, 1999; Hebert et al., 1998). Participants in the CG completed unrelated course work with no overarm throwing or QMD activities.

### Post-Testing

At the completion of the experimental and control interventions, participants rested for 10 min; then all three groups (VAG, VG & CG) completed post-testing, structured identically to the pre-testing. No feedback was provided in the post-testing.

After post-testing, participants completed a statement-based questionnaire to report their perceptions of their intervention and whether their motivation and confidence levels had been enhanced. As there was no previously validated questionnaire for this purpose, we based our own pilot questionnaire items on a literature review of similar questionnaires (Ferracioli et al., 2013; Koh & Khairuddin, 2004). The questionnaire we used consisted of 6-items. The first item identified participants’ previous formal throwing training with their non-dominant hand. The second item identified the level of confidence participants had to throw with their non-dominant hand prior to the intervention. Items three, four, and five assessed their perceptions of the intervention and item six assessed their thoughts about the importance of video analysis in the QMD process. The participants selected a response from a 5-point Likert scale (1 = *Strongly disagree* to 5 = *Strongly agree*). At the completion of the questionnaire all groups were instructed to refrain from non-dominant hand overarm throwing practice.

### Retention Testing

A retention test identical to pre- and post-testing was conducted three weeks after post-testing for all participants.

### Performance Assessment

To compare the effectiveness of the three interventions on throwing technique, we analyzed the throwing video footage according to Roberton and Halverson (1984) developmental levels (Table 1

**Table 1.** Modified Version of Roberton’s Developmental Levels.

Backswing action component	
Level 1	No backswing. Ball in the hand moves directly forward to release from the arm’s original position.
Level 2	Elbow and humeral flexion. Ball moves away from the target to a position behind or alongside the head by upward flexion of the humerus and elbow.
Level 3	Humeral lateral rotation. Ball moves away from the target by lateral rotation of the humerus in a position of 90° abduction.
Level 4	Circular, upward backswing. Ball moves away from the target to a position behind the head via a circular overhead movement with elbow extended, or a diagonal lift, or a vertical lift from the hip.
Level 5	Shortcut circular, downward backswing. Ball moves away from the target to a position behind the thrower via a circular, down and back motion, which carries the ball below waist height, followed by elbow flexion, at the end of the backswing the ball is forward of the outline of the thrower’s body (when viewed from behind).
Level 6	Circular, downward backswing. Ball moves away from the target to a position behind the thrower via a circular, down-and-back motion, carrying the hand below the waist, at the end of the backswing the ball is within the outline of the body (when viewed from behind).
Stepping action component	
Level 1	No step. The thrower throws from the initial foot position.
Level 2	Ipsilateral step. Thrower steps with the foot on the same side as the throwing hand.
Level 3	Short contralateral step. The thrower’s step with the opposite foot is half his or her standing height or less.
Level 4	Long contralateral step. The thrower’s step with the opposite foot is over half his or her standing height.
Follow-through action component	
Level 1	No follow-through. Arm movement stops when ball is released.
Level 2	Follow-through across the body. Throwing hand follows through across the body so that the whole hand disappears from sight when viewed from the side.
Trunk action component	
Level 1	No trunk rotation. Only the arm is active in force production. Sometimes the forward thrust of the arm pulls the trunk into a passive left rotation (assuming a right-handed throw).
Level 2	Block rotation. The hips and shoulders rotate away from the target and then towards the target simultaneously, acting as a unit or a block.
Level 3	Differentiated rotation, the hips precede the shoulders in initiating forward rotation. The thrower rotates away from the target then begins forward rotation with the hips then the shoulders begin rotating slightly after.
Humerus action component	
Level 1	Humerus oblique. The upper arm moves forward to ball release in a plane that intersects the trunk obliquely above or below the horizontal line of the shoulders.
Level 2	Humerus aligned but independent. The upper arm moves forward to ball release horizontally aligned with the shoulder, forming a right angle between humerus and trunk. When shoulders are front-facing, the upper arm and elbow have moved independently ahead of the outline of the body (as seen from the side) via horizontal adduction at the shoulder.
Level 3	Humerus lags. The upper arm moves forward to ball release horizontally aligned, when shoulders are front-facing, the upper arm remains within the outline of the body (as seen from the side).
Forearm action component	
Level 1	No forearm lag. The forearm and ball move steadily forward to ball release.
Level 2	Forearm lag. The forearm and ball appear to 'lag' (i.e., remain stationary behind the thrower). The largest lag occurs before the shoulders reach front-facing.
Level 3	Delayed forearm lag. The largest lag occurs at the moment shoulders are front-facing.

*Note.* These levels have been adapted from Roberton and Halverson (1984), Williams et al. (1998) and Beseler et al. (2021).

). Prior to beginning the study, to ensure inter-rater reliability, we completed two indepenedent reliability checks, using an expert researcher and a generalist trained primary teacher. The expert researcher with over 30 years’ experience categorized motor skills using Roberton’s (Roberton & Halverson, 1984) developmental levels to assist the first author in developing expert knowledge in the assessment of overarm throwing. To do this, the expert and the first author assessed throwing footage not included in the current study. Initially, these raters independently assessed the footage; they then met and discussed any ratings that were not identical. Assessment moderation continued until we achieved inter-rater reliability of 80% (Langendorfer & Roberton, 2002). After assessing 56 throws, the final inter-rater reliability results for each component were Backswing: 83%, Stepping: 85%, Follow-Through: 95%, Trunk: 85%, Humerus: 85%, and Forearm: 85%.

The first author then worked with a generalist trained primary teacher teaching as the designated PE teacher at a primary school to develop her assessment skills. After assessing 36 throws, the generalist primary teacher and the first author achieved inter-rater reliability results of Backswing: 81%, Stepping: 89%, Follow-Through: 97%, Trunk: 83%, Humerus: 81%, and Forearm: 83%. To confirm intra-rater reliability, the primary teacher then assessed another 20 throws one month apart. This teacher’s intra-rater reliability results were Backswing: 85%, Stepping: 95%, Follow-Through: 95%, Trunk: 85%, Humerus: 80%, and Forearm: 85%. Similar to a previous study (Haywood et al., 1991) the generalist primary teacher who had no involvement in testing, then completed the assessment of the throwing footage. The assessment of the six components were used to derive a summated scale ranging from 6 to 21 for each throw. The dependent variable, “throwing score,” was the total score of the three throws, which ranged from 18 to 63.

### Statistical Analysis

We used the Statistical Package for Social Sciences (SPSS for Windows, version 24.0) to perform a 3-factor (Group × Gender × Test) repeated measures analysis of covariance (3-way ANCOVA), with test as a within-subjects factor, gender and intervention group as between-subjects factors, covarying for age. Interactions between group, gender and test were also included in the model. We conducted independent sample t-tests to examine the difference between male and female throwing scores at various points in the experiment. All analyses had a *p* value of .05 for testing statistical significance.

The responses to the six survey questions were each cross tabulated against the intervention groups (VAG, VG, and CG). Because of small cell sizes, the five response categories were recoded into two categories (*Agree, and Disagree or Not sure*) with analysis by Fisher’s exact tests. Because the control group had not experienced an intervention, this group was excluded from analysis of responses regarding the interventions.

## Results

To ensure homogeneity of groups, we analyzed pre-test throwing scores for the three groups by a one-way ANOVA (see Table 2). No significant intervention group differences were found for the pre-test throwing scores, *F*(2,15) = .30, *p* = .970, partial ƞ^2^ = .001. The questionnaire results related to participants’ formal throwing training and confidence to throw with the non-dominant hand prior to the intervention were also analyzed to ensure homogeneity. The first statement survey item was, *I have had no formal throwing training using my non-dominant hand.* Every participant in each group agreed with this statement so homogeneity of prior training was assumed. The responses to the second survey statement, *I felt confident executing an overarm throw using my non-dominant hand prior to the intervention,* were analysed. Fisher’s exact test revealed the two groups self-reported equal confidence in throwing with their non-dominant hand at pre-testing. Given the pre-throwing score and non-dominant hand scores identified no differences, both groups were equivalent in non-dominant hand throwing experience at pre-testing.

**Table 2.** Throwing Score: Total Means (*M*) and Standard Deviations (*SD*).

Intervention group	Gender	*n*	Pre *M (SD)*	Post *M (SD)*	Retention *M (SD)*
VAG	Male	8	45.0 (4.1)	50.1 (3.5)	50.1 (4.8)
	Female	9	45.0 (4.2)	48.3 (3.8)	48.1 (5.3)
	Total	17	45.0 (4.0)	49.2 (3.7)	49.1 (5.0)
VG	Male	11	47.0 (5.6)	48.7 (6.3)	49.5 (6.2)
	Female	8	42.6 (2.9)	44.6 (3.9)	45.9 (3.7)
	Total	19	45.2 (5.0)	47.0 (5.7)	47.9 (5.5)
CG	Male	5	46.6 (5.0)	48.2 (2.8)	50.2 (4.2)
	Female	6	43.2 (4.4)	44.2 (4.2)	45.0 (5.4)
	Total	11	44.7 (4.8)	46.0 (4.1)	47.4 (5.3)

The within-subjects results revealed that the mean post-test throwing score values for all three groups combined (see Table 2 for descriptive statistics) were higher than the pre-test throwing scores, and the retention test throwing scores were higher than the post-test scores. However, these differences only approached statistical significance, *F* (2,80) = 2.825, *p* = .065, partial ƞ^2^ = .066. The between-subjects analysis found that the only statistically significant predictor of throwing score was gender. The male mean score of 48.1, adjusted for all other variables, including test, was significantly higher than the female mean score of 45.3, *F*(1,40) = 4.427, *p* = .042, partial ƞ^2^ = .100. An independent samples t-test indicated that males were higher at all testing points, with the difference in pre-test throwing scores approaching significance, *t*(45) = 2.0, *p* = .053, the male post-test throwing score significantly higher than the female post-test throwing score, *t*(45) = 2.4, *p* = .022, and the male retention test throwing score significantly higher than the female retention test throwing, *t*(45) = 2.3, *p* = .028. Throwing score was not significantly affected by intervention group, *F* (2,40) = .79, *p* = .463, partial ƞ^2^ = .038, and there was no significant interaction effect of intervention group and gender, *F*(2,40) = .46, *p* = .636, partial ƞ^2^ = .022.

The group by test interaction effect for combined gender was not significant, *F*(4,80) = 1.477, *p* = .217, partial ƞ^2^ = .069, and neither were the separate interaction effects for males, *F*(4,40) = 1.617, *p* = .19, partial ƞ^2^ = .139, and females, *F*(4,38) = .375, *p* = .83, partial ƞ^2^ = .038.

Although the group by test interaction effect was not statistically significant, an analysis of “simple effects” revealed different patterns of statistically significant changes over testing within the three groups. While this may appear inconsistent, it is not a contradiction. The two approaches address subtly different questions. The test of interaction examines whether the changes over time differed significantly between groups, while the simple effects tests indicated whether the changes within each group were significantly different from zero (Grace-Martin, n.d.). Situations can arise in which two effects are not significantly different from each other, but the larger effect is significantly different from zero while the smaller one is not. As such, we conducted the simple effects analysis for each group by adding Bonferroni-adjusted post-hoc analyses for the differences between test occasions. The VAG results showed a significant (mean difference = 4.253, *p* < .001) change in the pre- to post-test throwing scores and a significant (mean difference = 4.147, *p* < .001) change in pre- to retention scores. There was no significant change in the pre- to post-test scores for the VG (mean difference = 1.576, *p* = .238) or the CG (mean difference = 1.814, *p* = .398), but there was a significant change in the pre- to retention test scores in the VG (mean difference = 2.492, *p* = .011) and the CG (mean difference = 3.360, *p* = .011). There were no significant changes in throwing score from post to retention test scores in any of the intervention groups (VAG mean difference = .105, *p* = 1.00, VG mean difference = .916, *p* = .981, CG mean difference = 1.546, *p* = .664).

Levene’s tests were conducted to examine the equality of error variances. Results showed no significant departures from constant variance in pre-testing (*F*(2,20) *=* .405, *p* = .672), post-testing (*F*(2,20) = .274, *p* = .763), or retention testing (*F*(2,20) = .473, *p* = .630). Kolmogorov-Smirnov (K-S) and Shapiro-Wilk (S-W) analyses of residuals were used to test assumptions of normality; there were no significant departures from normality in pre-testing (K-S *p* = .200, S-W *p* = .418), post-testing (K-S *p* = .200, S-W *p* = .448), and retention testing (K-S *p* = .200, S-W *p =* .270).

The questionnaire responses were examined to determine the participants’ perceived impact of the interventions on throwing ability, QMD skills, the importance of video analysis technology in the QMD process, and the impact video analysis had on enjoyment level when learning to throw with their non-dominant hand. The VAG (94.1%) and VG (100%) both responded positively about the impact their respective interventions had on their throwing ability. The Fisher’s exact test revealed no significant group effect, *p* = .472*.* Both VAG and VG participants believed their respective interventions helped improve their non-dominant hand throwing.

The VAG (82.4%) and VG (89.5%) responded positively about the impact that the interventions had on their confidence to perform QMD. The Fisher’s exact test revealed no significant group effect, *p* = .650. Both VAG and VG participants believed their respective interventions helped them perform QMD on their partner’s overarm throwing.

Only the VAG experienced video replay in their intervention; however, all participants experienced multiple video analysis sessions in their fundamental movement course prior to this study. As such, the VG provided experiential responses about the importance of video replay. The Fisher’s exact test revealed no significant group effect, *p* = .264, indicating that video replay was similarly essential for both groups. Results also indicated that VG (94.7%) participants found their intervention more enjoyable and engaging than did the VAG (64.7%) participants. The Fisher’s exact test revealed a significant group effect, *p* = .037.

## Discussion

Our purposes in this study were to determine whether a single session peer-teaching intervention could improve short-term non-dominant hand overarm throwing performance among pre-service PE teachers and to examine what perception these participants had of the interventions. Important findings from the current study were the immediate improvements made by participants with access to video analysis during their throwing intervention, and the perceived improvements that both peer-teaching interventions had on participants’ throwing and QMD skills.

### Throwing Performance

In this study, throwing technique improved for all three groups, with VAG the only group to show significant pre- to post-test improvement; yet there was no significant difference between the mean scores of the three groups at post- and retention testing. Thus, our hypotheses that the VAG group would throw with more advanced technique in the post- and retention testing than the VG group, and that the VG group would throw with more advanced technique than the CG group were not supported. This may be the result of a ceiling effect. Since the participants were acquiring a skill previously learned with their dominant hand, they may have achieved a performance level that made it difficult to see distinct improvements after further training.

The immediate VAG improvements were similar to Robles’ (2013) finding that the presentation of verbal and video feedback to students learning the grab start swimming dive was more effective than receiving verbal feedback alone. A prominent explanation of the immediate improvements in both Robles’ (2013) study and ours was that video feedback provided learners with visual movement information to compare to correct form that was used to detect errors and modify ensuing performances (Menickelli et al., 2000). The video feedback may have increased both observers’ and throwers’ observational powers and abilities for qualitative analysis (Koh & Khairuddin, 2004). Enhancing feedback with videos facilitated adaptations during practice (Potdevin et al., 2018), and allowed efficient skill acquisition.

A second, more speculative, explanation for this “faster” acquisition is that the VAG intervention involved more explicit motor learning that led to higher conscious awareness of how the throw should be performed (Kleynen et al., 2014). More opportunity to visually critique another thrower’s technique in the VAG intervention may have generated additional explicit knowledge, encouraging learners to increase their attentional control to their movements. This greater internal focus of attention (Kal et al., 2018) may have led to more rapid performance improvements (Rendell et al., 2011), but Masters (1992) reported that internal focus of attention may deteriorate under pressure. Throwing with the non-dominant hand was the skill chosen because the task novelty accounted for participants’ past throwing experience and helped participants experience the type of feelings their students might experience when acquiring new motor skills.

### Gender Differences

We found that males had more advanced throwing technique than females initially and throughout the study, irrespective of their group membership. As such, our hypothesis that males would throw with more advanced technique than females was supported, a finding that is consistent with prior studies that examined gender differences in throwing both quantitatively (e.g., throwing velocity and distance) and qualitatively (Gromeier et al., 2017; Johnson et al., 2020; Lorson et al., 2013; Schott & Getchell, 2021). On average, males have been found to throw with more accuracy, velocity, and advanced technique than females at all ages. Of note, however, we found no differential intervention influence across males and females.

### Impact on Perceived Throwing Ability

We found that participants in both VAG and VG believed that their respective interventions had helped them improve non-dominant hand throwing, indicating our hypothesis of an advantage for VAG participants in this regard was not supported. These positive attitudes from participants were similar to the findings of other investigators of the effectiveness of peer-teaching (Ensergueix & Lafont, 2010; Johnson & Ward, 2001). Furthermore AlShareef et al. (2019) found that students’ perceptions of reciprocal peer-teaching were similar to their perceptions of faculty teaching, with an overwhelming majority of students having reported professional and personal benefit from this instructional approach. Furthermore, Ensergueix and Lafont (2010) found similar results with higher self-efficacy of table tennis skills among recipients of peer teaching compared to participants who practiced individually without peer tutoring. Similarly, Ferracioli et al. (2013) found that participants in both the VAG and VG groups thought that their respective interventions were effective in helping to improve swimming performance, with similar motivational effects during the five-day breaststroke learning process.

### Impact on Perceived QMD Ability

Our questionnaire results indicated that participants in both VAG and VG groups perceived their ability to analyze the overarm was effective. Our hypothesis of a VAG group advantage relative to the VG group was not supported, in contradiction to findings from two research teams that examined the integration of video analysis into a PE setting (Koh & Khairuddin, 2004; O'Loughlin et al., 2013). O’Loughlin et al. (2013) found primary school children’s performance assessment perspectives to have been aided by video footage in PE, when compared to traditional teacher feedback, and Koh and Khairuddin (2004) found that video analysis better improved learners’ observational powers. One possible explanation for these contradictory finding is the varied length of the interventions in these studies. Our method involved one 20-min intervention session, while Koh and Khairuddin (2004) studied a 9-week gymnastics intervention, and O’Loughlin et al. (2013) studied a 10-week basketball intervention. Perhaps the perceived QMD benefits of video analysis are not immediately evident to students.

### Importance of Video Analysis

Both VAG and VG participants reported that video analysis was essential in the QMD process. Thus, our hypothesis that the VAG would report higher importance of the video analysis technology to the QMD process compared to the VG was not supported. While only the VAG experienced video analysis in the intervention, all participants had completed familiarization sessions during their fundamental movement course to ensure they were not distracted by viewing video footage of themselves on screen for the first time (Beseler & Plumb, 2019; Darden, 1999; Darden & Shimon, 2000). These experiences may have been enough for the VG to develop opinions about the importance of video analysis. The value that participants in this study placed on video analysis is consistent with previous findings that have video analysis assists participants to recognize strengths and weaknesses (Weir & Connor, 2009), helps participants understand cues (Kretschmann, 2017), enables performance assessment (O’Loughlin et al., 2013), and enhances observational and QMD skills (Koh & Khairuddin, 2004).

### Enjoyment Level

Participants who experienced the verbal intervention reported enjoying and engaging with it more than did those in the video analysis intervention. Thus, our hypothesis that the VAG would report greater enjoyment than the VG was not supported. These findings contradict prior video analysis research indicating that learners better enjoyed and were better motivated by watching video replays of their performances (Ferracioli et al., 2013; Koh & Khairuddin, 2004). Again, the number of intervention sessions involved in these respective studies may be a basis for these different findings. In contrast to our results with a single session, other investigators used five (e.g., Ferracioli et al., 2013; Palao et al., 2013) or 16 sessions (e.g., Casey & Jones, 2011). Perhaps the greater number of sessions allowed participants to become accustomed to the technology and enjoy the sessions more, while our participants may have felt overwhelmed by just a single session (Obrusnikova & Rattigan, 2016).

### Limitations and Directions for Future Research

Other researchers found that students who improved performance because of video analysis were more motivated and engaged by the feedback, often leading to more practice inside and outside of PE classes (O'Loughlin et al., 2013). Although speculative, the immediate performance improvements for participants in the VAG group from a single session in our study led to motivational benefits that enhanced learner’s perceived success and continued practice (Knudson, 2013), but we did not gather this follow-up data. There is scope for further research to examine whether video analysis improves students’ motivation levels in these ways. Future investigators could also explore whether benefits of video analysis in a peer-teaching setting apply to both primary school and secondary school learners. If school aged learners can show immediate motor skill improvements after a single 20-min session, it could allow the transfer of these newly acquired skills to a different context, such as game based activities in their PE classes, recreational activities with their friends, and/or more formal, organized sporting activities (Horn et al., 2007). Ultimately, we may be able to help these learners develop their fundamental motor skills, in the process increasing the likelihood of subsequent healthy physical activity (Barnett et al., 2013). Given that our single 20-min session did not lead to strong group differences at retention testing, future researchers might extend the number of peer-teaching sessions to determine if more robust improvements can then be achieved.

## Conclusion

Our findings in this study have shown that pre-service PE teachers working in a peer-teaching setting for a single 20-min session can show immediate improvements in their non-dominant hand overarm throwing techniques if video analysis feedback is used during practice. However, gains were comparable in CG and VG groups at retention testing. In light of the immediate improvements we observed from adding video analysis technology, we recommend that PETE programs incorporate peer-teaching/video analysis sessions into fundamental movement courses. This might help pre-service PE teachers develop the FMS more quickly. As in primary and secondary PE, the crowded curriculum of PETE programs makes it difficult for pre-service teachers to fully develop all FMS. Video analysis sessions could facilitate pre-service PE teachers’ quicker development of FMS proficiency, making them more effective teachers through their ability to proficiently demonstrate the skills they are teaching (Baghurst et al., 2015; Pulling & Allen, 2014) and increasing their confidence in implementing these approaches in primary and secondary school PE classes across the world. Considering the rapid and lasting growth of learning via digital technology, arming PE teachers with these technological skills might also further leverage their ability to engage young learners (Casey et al., 2017).
